# Comparison of Sudden Sensorineural Hearing Loss with Tinnitus and Short-Term Tinnitus

**DOI:** 10.1155/2021/6654932

**Published:** 2021-04-20

**Authors:** Fang Qi, Liang Chaoqun, Yan Lin, Yang Jianming

**Affiliations:** Department of Otorhinolaryngology, Head and Neck Surgery, The Second Affiliated Hospital of Anhui Medical University, Hefei, Anhui Province, China

## Abstract

**Objective:**

As one of the common symptoms of sudden sensorineural hearing loss (SSH), tinnitus seriously affects the life and work of SSH patients. The present study is aimed at exploring whether SSH can receive acoustic therapy and the factors that affect the efficacy of SSH acoustic therapy.

**Methods:**

A total of 162 patients were outpatients and inpatients, 86 were SSH, and 76 were short-term tinnitus (STT). Both groups received pure tone audiometry, tinnitus matching, and residual inhibition test (RI). The Tinnitus Handicap Inventory (THI), visual analog scale with respect to tinnitus loudness (VAS), and RI in each group were evaluated. The effects of age, degree of hearing loss, and tinnitus course on the efficacy of SSH acoustic therapy were also evaluated.

**Results:**

In the comparison of RI, THI, and VAS, there was no difference between SSH and STT (*P* > 0.05). SSH patients with mild hearing loss showed better acoustic therapy efficacy compared with SSH patients with severe hearing loss (*P* < 0.05), but there is no statistical difference in age and the course of tinnitus (*P* > 0.05).

**Conclusion:**

The present study showed that SSH may improve tinnitus symptom through receiving acoustic therapy and SSH patients with mild hearing loss can get better acoustic therapy effects.

## 1. Introduction

Sudden sensorineural hearing loss (SSH) is an unexplainable sensorineural hearing loss with onset in less than 72 hours, and the estimated incidence is between 5 and 20 per 100,000 people per year [1, 2]. Tinnitus refers to a subjective feeling that the patient feels there is sound in the ear or brain when there is no corresponding sound source and electrical stimulation source in the surrounding environment [3]. Tinnitus is also one of the common concomitant symptoms of SSH, with an incidence of 78.2-91.0%; some SSH patients have their hearing recovered or improved after treatment, but the tinnitus still exists for a long time or intermittently, which seriously disturbs the work and life of the SSH patients [4]. Recent studies have shown that tinnitus patients with a shorter course are more affected by tinnitus than those with a longer course, which may be related to the patient's adaptation to tinnitus [5].

The acoustic therapy of tinnitus, a method suppressing tinnitus through using sound to change the perception and response of tinnitus, includes special flame wave music therapy centered on tinnitus frequency; neuromusical therapy; neural desynchronization modulation and modulate the sound [6–8], etc. The results of various studies on the efficacy of acoustic therapy vary greatly, ranging from 21 to 67%. In acoustic therapy, in order to achieve the curative effect of acoustic therapy, it is necessary to detect tinnitus (tinnitus main tone matching, loudness matching, residual inhibition test (RI), etc.) [4]. Most studies have shown that the efficacy of tinnitus patients with a good tinnitus match and a positive tinnitus RI can reach 75-92% [6]. This means that patients with positive RI are recommended for acoustic therapy. Studies have compared from 156 cases that acoustic therapy has a certain effect on tinnitus patients with normal hearing and hearing loss, and there is no statistical difference in efficacy between the two groups [9]. SSH is also a kind of tinnitus accompanied by hearing loss, so whether SSH can be treated with sound is worth studying.

Cochlear hair cell damage is one of the possible pathogenesis of tinnitus, because the tinnitus frequency of most patients tends to appear at the frequency of hearing loss. Previous studies have suggested that SSH patients cannot elicit distortion product otoacoustic emissions (DPOAE) and deemed SSH may damage cochlear hair cells [10]. Therefore, some studies suggest that SSH and tinnitus may have similarities in pathogenesis and lesions. The previous study compared the drug treatment effects of SSH patients and deemed that the better the hearing curative effect of SSH patients accompanied by tinnitus, the better the curative effect of tinnitus, which further suggests that the cochlear hearing loss site of SSH is related to the site of tinnitus loss [10]. Therefore, we speculate that the degree of SSH hearing loss may be closely related to tinnitus.

In studying the factors affecting the prognosis of SSH, which has been reported that adolescents and elderly patients have a poor prognosis and believed that the SSH of the elderly people with high blood pressure, diabetes, cerebrovascular disease, and other systemic diseases causes severe hearing loss at the onset, the SSH of adolescents is mostly caused by a history of viral infection [11–13].

Acoustic therapy is a widely used method for the treatment of tinnitus, but there is a lack of research on SSH in previous studies. The present study aimed to whether SSH can receive acoustic therapy and the factors that affect the efficacy of acoustic therapy for SSH.

## 2. Methods

### 2.1. Participants

This study included a total of 162 data files of tinnitus patients between the ages of 18 to 88. All participants were outpatients and inpatients of our department during 2018.5-2020.8. These patients were divided into 2 groups, 86 participants were SSH patients, and 76 participants were short-term tinnitus patients (STT). The eligibility criteria for SSH patients included sensorineural hearing loss that develops within 72 hours and hearing loss of more than 30 dB in 3 adjacent frequencies. All SSH patients must be accompanied by unilateral or bilateral tinnitus. The eligibility criteria for STT patients were that the course of tinnitus does not exceed 1 month and pure tone audiometry does not reveal hearing loss. Accorded to medical history, symptoms, and otological examinations (electrical otoscope, auditory function, and computer tomography), all tinnitus patients excluded middle ear diseases that may cause tinnitus, otitis media, Meniere's disease, and other ear diseases that may cause tinnitus. Excluded patients are with systemic diseases that may cause tinnitus, such as high blood pressure and diabetes. Patients were required to have the ability of the communicating for examination and treatment (such as medical history inquiry). All participants verbally agreed to use their medical data. The study was approved by the Ethics Committee of the Second Hospital of Anhui Medical University: YJ-YX2019-039.

### 2.2. Pure Tone Audiometry and Tinnitus Matching

All patients need to have pure tone audiometry and tinnitus matching. The hearing tests involved in this study all use tinniest comprehensive tinnitus diagnosis and treatment equipment. The equipment conformed to the standard GB/T-16403. The test place was a soundproof shielded room and the environmental noise < 20 dB. All of the pure tone audiometry and tinnitus matching were completed by the same technical personnel.

Tinnitus matching is as follows:

1. *Tinnitus Frequency Matching*. The technical personnel sends a pure tone to the tinnitus ears (if bilateral tinnitus, the comparative severe side shall prevail) through tinniest equipment and gave the patients 3 adjacent test tones (e.g., 2, 4, and 8 kHz) (at the upper threshold of the standard frequency 5 dB) to let the patients choose the stimulus with the frequency closest to the tinnitus (e.g., 4 kHz is the closest). Then, narrowed the frequency range through centering on the previous tone and selected another 3 adjacent test tones to repeat the above steps (such as 3, 4, and 5 kHz) until the given pure tone frequency was the patient's tinnitus frequency. The final pure tone frequency was set as the main tone of tinnitus
2. *Tinnitus Loudness Matching*. On the basis of the final pure tone frequency, gradually increase the loudness in units of 1 dB to just cover tinnitus, and this loudness is the tinnitus loudness

The degree of hearing loss: according to the average value of hearing loss in pure tone audiometry, the hearing loss was divided into mild (25-40 dB), moderate (41-55 dB), moderate to severe (56-70 dB), and severe (>70 dB).

Age group: according to the United Nations World Health Organization (2013) standards, patients ≤ 44 years old are divided into the youth group, and >44 years old are the middle-aged and elderly group.

### 2.3. Outcomes

Tinnitus Handicap Inventory (THI): THI included 25 choice questions about function, emotion, and disaster. Each question provided 3 options: “No,” “Sometimes,” and “Always,” and the score for each option is 0, 2, and 4. The severity of tinnitus was judged through the total score of THI. The higher the score, the more serious the tinnitus and the greater the impact on the patients. There are THI tests for all patients before and after RI.

Visual analog scale with respect to tinnitus loudness (VAS): the VAS scale included loudness (“How loud is your tinnitus?”) and annoyance (“How stressful is your tinnitus?”). Both items were evaluated on a scale from 0 (no tinnitus/no stress) to 10 (extremely loud/extremely stressful). The VAS tests for all patients before and after RI.

Residual inhibition test (RI): given the patients a sound 10 dB higher than tinnitus lasted for 1 min. Recorded the changes in the patient's tinnitus after the sound stimulation stop: positive (Type I) means the tinnitus disappears completely; partially positive (Type II) means the tinnitus loudness is reduced for a period of time or the sound quality changes for a period; negative (Type III) means no change in tinnitus. RI is required for both groups.

The THI, VAS, and RI results between the two groups are compared. SSH patients according to ages, the degree of hearing loss, and the duration of the SSH to further compare RI, THI, and VAS are grouped.

### 2.4. Statistical Analyses

The data were analyzed with the professional SPSS software 23.0.

For the comparison of general conditions between groups, such as age, course of tinnitus, loudness of tinnitus, frequency of tinnitus, the Mann-Whitney *U* test, and the *T* test were used. The chi-square test was used to compare gender and unilateral-bilateral tinnitus between two groups.

The Wilcoxon test and the Kruskal-Wallis test were used to compare VAS and THI in groups. The chi-square test was used to compare RI results among groups.

## 3. Results

### 3.1. Comparison of SSH and STT Groups

The basic information of the SSH group and the STT group is shown in Table 1. In the unilateral and bilateral tinnitus comparison, there were statistical differences between the SSH group and the STT group (*P* < 0.001). The pure tone average (*P* < 0.001) and the loudness of tinnitus (*P* < 0.001) in the SSH group were significantly higher than the STT group. There was no statistical difference in the comparison of age, gender, course of disease, and frequency of tinnitus (*P* > 0.05).

In the comparison of acoustic therapy indicators, THI before RI, THI after RI, VAS before RI, VAS after RI, and RI were not statistically different between SSH and STT groups (*P* > 0.05).

### 3.2. Comparison of SSH in Different Age Groups

In the comparison of SSH patients ≤ 44 years old and >44 years old, there was no statistical difference in VAS, THI, and RI (*P* > 0.05) (Table 2).

### 3.3. Comparison of SSH in Different Degrees of Hearing Loss

The THI (*P* < 0.001), VAS (*P* < 0.001), and positive rates of RI (*P* = 0.003) in SSH patients with mild hearing loss were significantly higher than moderate, moderate to severe, and severe SSH patients.

### 3.4. Comparison of SSH in Different Tinnitus Course

In the comparison of SSH tinnitus course ≤ 1 W and >1 W, there was no statistical difference in VAS and RI (*P* > 0.05). The THI value of SSH patients with tinnitus duration ≤ 1 W is lower than that of SSH patients > 1 W (*P* = 0.041) (Table 2).

## 4. Discussion

The present study is aimed at verifying the hypothesis that SSH patients with tinnitus can receive acoustic therapy. The study had shown that there is no difference between SSH and SST in the comparison of acoustic therapy indicators. The retrospective study we designed involved a total of 162 cases in outpatients and inpatients and compared factors such as RI, VAS, and THI. Except for SSH in the pure tone average, loudness of tinnitus, and unilateral-bilateral tinnitus, there was no difference between SSH and STT. In order to further study the factors that may affect the curative effect of tinnitus in SSH patients, we conducted comparisons according to age, hearing loss degree, and tinnitus course. The study has shown that SSH patients with mild hearing loss show better acoustic therapy efficacy compared with SSH patients with severe hearing loss, but there is no statistical difference in age and the course of tinnitus.

The current assumption of the underlying mechanism is that RI is produced by neuronal changes in excessive activity at peripheral or central levels caused by hearing loss following acoustic stimulation in the deafferent regions. Previous studies have shown that particular characteristics of the acoustic stimulus targeting tinnitus and hearing lesions are known to influence the depth and duration of RI. Terry et al. (1983) observed that the maximum RI time increases in a logarithmic fashion with increasing stimulus duration. Moreover, it is known that an acoustic stimulation resembling the hearing loss that often coincides with the tinnitus spectrum induces RI more effectively. However, although hearing loss is one of the main causes of tinnitus, it is not a necessary condition. In addition to hearing loss, tinnitus chronicity has been shown to be a contributory factor in the pathology of tinnitus. Vanneste et al. (2011) demonstrated a change in activity in several brain areas and in functional connectivity between auditory and nonauditory brain structures over time. Other studies observed a correlation of tinnitus chronicity with frontostriatal connectivity and thalamic functional connectivity to different brain regions in normal hearing subjects. In addition, in previous studies, changes in neuronal activity in both auditory and nonauditory brain areas were observed during RI.

The previous study found that distortion product otoacoustic emissions (DPOAE) showed “mountain-shaped” drop in the low-frequency region and above 5 KHz in 13 tinnitus patients with normal hearing [14]. This suggested that tinnitus patients with normal hearing threshold may have cochlear damage and all tinnitus patients may have hidden hearing loss. Some studies believed that some SSH patients cannot elicit DPOAE, which may be due to the damage of cochlear hair cells in SSH patients [15]. These conclusions suggested that tinnitus patients and SH patients may have similar pathology and pathogenesis, which can also explain that why there is no statistical difference between SSH and STT in the results of RI, THI, and VAS in this study. All SSH patients have hearing loss, so the cochlear hair cell damage of SSH may be more serious than the tinnitus with normal hearing, which may be the reason why the tinnitus loudness of the SSH was higher than STT. Many studies had shown that SSH usually unilateral and bilateral is rare, which can explain why SSH and STT have a significant difference in unilateral and bilateral comparison.

Many studies believed that SSH patients over 55 years of age have a poor prognosis due to systemic diseases such as hypertension, diabetes, and cerebrovascular disease. Studies had shown that the plasma viscosity, whole blood viscosity, and red blood cell aggregation index of elderly SSH patients are higher than those of normal patients with SSH. This indicated that the blood of elderly SSH patients is in a state of hypercoagulability, which slows the blood flow in the peripheral arteries [13]. Moreover, hyperlipidemia can degenerate red blood cells and reduce their ability to carry relevant nutrients. This low flow rate and low perfusion cause inner ear tissue ischemia and hypoxia, damaged hair cells, resulting in a worse prognosis for SSH. Most young patients with SSH have a history of fever, cold symptoms, or increased viral antibody titer before the onset, and the studies reported that young patients have a poor prognosis. However, the present study showed that there is no statistical relationship between tinnitus of SSH and age.

The frequency range and degree of hearing loss in SSH are different, and the pathogenesis is also different. Studies had speculated that low-frequency hearing loss may be caused by membrane labyrinth hydrops or local blood supply disorder of the spiral ligament, and high-frequency hearing loss may be caused by outer hair cell damage (hearing loss is at least 50 dB) or inner hair cell damage (hearing loss is at least 60 dB); severe deafness may be caused by vascular embolism or thrombosis of the common cochlear artery or the axial spiral artery, resulting in irreversible and serious damage to the hair cells of the entire cochlea. Therefore, the more severe the hearing loss, the worse the prognosis may be. In the present study, the hearing loss degree of SSH was closely related to the indicators of acoustic therapy, and 1patients with mild hearing loss had a higher RI positive rate.

Most scholars believed that the treatment effect and prognosis of SSH are closely related to the time of initial diagnosis. The earlier the initial diagnosis, the better the prognosis. But in the present study, we did not find similar differences in the acoustic therapy of SSH tinnitus [16].

In tinnitus matching, the matching rate affects the efficacy of acoustic therapy, the matching rate of high-frequency tinnitus is lower and the low-medium-frequency tinnitus is higher. This may be related to the plasticity of the patient's auditory center [17]. After repeated sound stimulation, the center's perception of sound loudness can be adaptively adjusted. Therefore, accurately matching the tinnitus frequency can accurately increase the excitability of tinnitus-related nerves and reduce the inhibitory effect [18]. Some studies believed that the dizziness in SSH patients indicates a poor prognosis. Therefore, the tinnitus frequency and dizziness of SSH may also affect the efficacy of acoustic therapy. Due to the limitation of sample size and sample case information, the research cannot be carried out.

## 5. Conclusion

Acoustic therapy is a noninvasive, safe, simple, and easy treatment; the present study showed that SSH may improve tinnitus symptom through receiving acoustic therapy and SSH patients with mild hearing loss can get better acoustic therapy effects. As a new treatment strategy, it needs to be further confirmed in the follow-up multicenter, large sample systematic observation.

## Figures and Tables

**Table 1:** The basic information of SSH and STT (*n* = 162).

	SSH	STT	*P*
Number of patients	86	76	
Gender (male/female)	51/35	35/41	0.092
Age (years) (SD)	40.59 (1.61)	34.41 (1.96)	0.062
Course of tinnitus (days) (SD)	14.20 (1.07)	14.89 (1.19)	0.902
Unilateral tinnitus/bilateral tinnitus	81/5	42/34	<0.001
Left ears/right ears	42/49	58/52	0.354
Frequency of tinnitus (HZ) (SD)	2931.44 (263.84)	3870.95 (338.05)	0.060
Loudness of tinnitus (dB) (SD)	46.98 (2.25)	22.01 (1.87)	<0.001
Pure tone average (SD)	57.30 (1.91)	17.31 (0.50)	<0.001
THI before RI (SD)	44.55 (2.60)	41.09 (2.50)	0.417
THI after RI (SD)	34.28 (2.62)	33.37 (2.38)	0.949
VAS before RI (SD)	5.41 (0.20)	4.75 (0.21)	0.051
VAS after RI (SD)	4.05 (0.25)	3.75 (0.23)	0.511
RI: completely positive/partially positive/negative	14/37/35	4/39/33	0.079

SD: standard deviation; SSH: sudden sensorineural hearing loss group; STT: short-term tinnitus group; THI: the Tinnitus Handicap Inventory; VAS: visual analog scale with respect to tinnitus loudness; RI: residual inhibition.

**Table 2:** The comparison of THI, VAS, and RI of SSH.

		THI (SD)	VAS (SD)	RI: completely positive/partially positive/negative
Age	≤44 years	9.53 (2.09)	1.29 (0.21)	9/22/18
	>44 years	11.24 (2.65)	1.54 (0.34)	5/15/17
	*P*	0.879	0.893	0.658

Degree of hearing loss	Mild	19.74 (4.89)	2.58 (0.52)	6/11/2
	Moderate	10.75 (2.19)	1.63 (0.33)	2/16/6
	Moderate to severe	10.95 (3.59)	1.30 (0.33)	5/6/9
	Severe	1.35 (0.95)	0.26 (0.16)	1/4/18
	*P*	<0.01	<0.01	0.003

Tinnitus course	≤1 W	7.17 (2.57)	1.09 (0.27)	6/11/18
	>1 W	12.39 (2.10)	1.61 (0.26)	8/26/17
	*P*	0.041	0.135	0.170

SD: standard deviation; SSH: sudden sensorineural hearing loss group; THI: the difference of THI before and after RI; VAS: the difference of VAS before and after RI; RI: residual inhibition.

## Data Availability

The datasets used and/or analyzed during the current study are available from the corresponding author on reasonable request.
